# Emerging Concepts Promising New Horizons for Marine Biodiscovery and Synthetic Biology

**DOI:** 10.3390/md13052924

**Published:** 2015-05-13

**Authors:** F. Jerry Reen, José A. Gutiérrez-Barranquero, Alan D. W. Dobson, Claire Adams, Fergal O’Gara

**Affiliations:** 1 BIOMERIT Research Centre, School of Microbiology, University College Cork—National University of Ireland, Cork, Ireland; E-Mails: j.reen@ucc.ie (F.J.R.); j.gutierrez@ucc.ie (J.A.G.-B.); c.adams@ucc.ie (C.A.); 2 School of Microbiology, University College Cork—National University of Ireland, Cork, Ireland; E-Mail: a.dobson@ucc.ie; 3 School of Biomedical Sciences, Curtin University, Perth WA 6845, Australia

**Keywords:** marine bioactives, lantibiotics, signaling molecules, peptides, mimetics, quorum sensing, quorum quenching, motif/domain

## Abstract

The vast oceans of the world, which comprise a huge variety of unique ecosystems, are emerging as a rich and relatively untapped source of novel bioactive compounds with invaluable biotechnological and pharmaceutical potential. Evidence accumulated over the last decade has revealed that the diversity of marine microorganisms is enormous with many thousands of bacterial species detected that were previously unknown. Associated with this diversity is the production of diverse repertoires of bioactive compounds ranging from peptides and enzymes to more complex secondary metabolites that have significant bioactivity and thus the potential to be exploited for innovative biotechnology. Here we review the discovery and functional potential of marine bioactive peptides such as lantibiotics, nanoantibiotics and peptidomimetics, which have received particular attention in recent years in light of their broad spectrum of bioactivity. The significance of marine peptides in cell-to-cell communication and how this may be exploited in the discovery of novel bioactivity is also explored. Finally, with the recent advances in bioinformatics and synthetic biology, it is becoming clear that the integration of these disciplines with genetic and biochemical characterization of the novel marine peptides, offers the most potential in the development of the next generation of societal solutions.

## 1. Introduction

The ocean has proven to be a vast reservoir of resources for human consumption and utilization for millennia. A natural ecosystem to many kingdoms of organisms, the marine environment is host to producers of a rich tapestry of compounds and molecules with huge therapeutic and industrial potential. The exponential advances in new technologies and engineering capacity have opened up the marine ecosystem to scientific exploration. As a result, new sources of the next generation of therapeutics, biocatalysts and natural products continue to emerge.

The source of bioactive material in the oceans has been diverse. Algae and other higher order marine life have received considerable attention, with associated microbial communities gaining prominence. Bioactive compounds from these sources have ranged from anti-coagulants to anti-cancer and more recently to the next generation of antimicrobial compounds [1,2,3,4,5,6,7,8]. In spite of considerable challenges, some of these bioactive compounds have made it to the market, providing a roadmap for future translational efforts (reviewed in [9]). Examples of those which have reached the market in the pharmaceutical sector are Trabectedin (ET-743, Yondelis^®^), a novel marine antineoplastic alkaloid for the treatment of advanced soft tissue sarcoma; Lovaza^®^ (former Omacor), an anti-hypertriglyceridemia drug; Ziconotide (Prialt^®^), a synthetic derivative of a naturally occurring ω-conotoxin for the management of severe chronic pain; and Eribulin mesylate (Halaven^®^), a simplified macrocyclic ketone analogue of Halichondrin B from the marine sponge *Halichondria okadai* with anti-cancer properties [9]. The cosmetics industry has seen the introduction of Abyssine^®^, Resilience^®^, SeaCode^®^, and RefirMAR^®^, all of which are marine derived and largely based on extracellular matrices such as exopolysaccharide (EPS) [9]. Apart from small molecular therapeutics, marine biodiscovery programs are also delivering new industrial enzymes with improved bioactivity, leading the movement towards green chemistry, while the identification of cell-cell communication compounds in the marine system is providing new insights into cell-cell signaling in the microbial pathogens [10,11].

**Figure 1:**
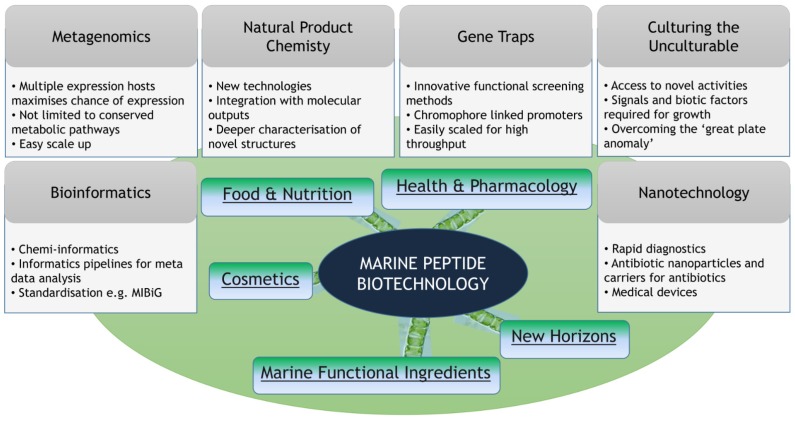
**Biotechnology applications of marine peptides**. The enormous potential for application of marine peptides has begun to be realized in recent years. Developments, both technological and societal, across a wide spectrum of industries have led to the use of peptides in previously unforeseen products. As technologies continue to develop, and pending our capacity to harvest the rich reservoirs of rare and novel bioactive peptides, the market need for these molecules is likely to continue its growth trajectory.

Therefore, having established the potential of the marine ecosystem to deliver these societal advances and solutions, the challenge remains to optimize our capacity to identify and mine them. Some success has already been achieved through culture independent approaches, using metagenomics to translate the genetic blueprint into active compounds [12,13,14,15]. In addition, new initiatives are ongoing to improve the culturability of marine isolates, thus enhancing our capacity to produce the novel compounds that are urgently needed [16,17,18,19,20,21,22,23,24]. However, perhaps a greater challenge remains the integration of molecular and chemical technologies to maximize our efficiency in this field. Individually, advances in the chemical and genetic sciences have provided significant improvements in the technologies available to identify and isolate new compounds and bioactivities from a broad spectrum of environmental ecosystems. Together, however, these technologies can be far more powerful, and there has been considerable recent interest in the integration of chemical and genetic technologies with the added power and discrimination offered by informatics. Improved discovery and mining of marine bioactivities has fed into the development of new and innovative solutions across a broad spectrum of areas including anti-inflammatories, antibiotics, anti-coagulants and anti-infectives (Figure 1 and Table 1). The importance of continued advances in the isolation and characterization of these activities is highlighted by the emergence of resistance mechanisms to most conventional therapies.

**Table 1:** Marine Peptide Biotechnology.

Marine Sector	Application
Functional Ingredients	Algae and biofuels
	Seafood byproducts
Food and Nutrition	Food safety and quality
	Aquaculture
	Bioprospecting
	Nutrition
	Product stability
	Seafood and health
	Nutraceuticals
New Horizons	Photography
	Textiles
	Leather
	Electronics
Cosmetics	Biofilms
	Antioxidants
	Dispersants
	Emulsifiers
	Anti-ageing

Marine peptides have emerged as primary candidates for therapeutic development in recent years [25]. This field has seen considerable advances in the integration of technologies and the extraction and exploitation of marine peptides has begun to deliver on its promise. The potential diversity of marine peptides is almost infinite, hidden in an inactive form in the native protein, until released through proteolysis. Novel lantibiotics from the marine ecosystem provide increased activity against multi-drug resistant pathogens, while peptide and small molecule mimetics have shown potency against microbial communities called biofilms. Maximizing our ability to harvest these activities will require the integration of functional and informatics approaches. Furthermore, as protein characterization and the degree of available structural information continue to advance, targeted motif-analysis could provide new insights into the distribution of key activities, providing a focus and platform for future functional screens. In this paper we describe some recent advances in this area and offer insights into where the next generation of integrative technologies for peptide mining will spring from.

## 2. Bioactive Marine Peptides

Peptides show great pharmaceutical potential as active drugs and diagnostics as well as being excellent functional excipients in drug delivery systems to overcome tissue and cellular membrane barriers. For that reason, the extraction and utilization of marine peptides has found new applications in recent years, particularly when allied with technological developments in other disciplines.

### 2.1. Antimicrobials/Lantibiotics

With the worldwide resistance to most known classes of current antibiotics reaching critical levels, marine environments and in particular marine microorganisms, which often experience extreme and stressful environments, are becoming a rich source of novel antimicrobial peptides (AMPs) [26,27,28]. Microbial AMPs are classified into ribosomally synthesized (bacteriocins) or non-ribosomally synthesized peptides, the products of non-ribosomal peptide synthase (NRPS) associated secondary metabolism.

The ribosomally synthesized or bacteriocin class of AMPs are recently gaining particular interest as alternative antibiotics. Bacteriocins are small peptides, which usually function by altering the membrane of target bacteria causing cell death. While generally bacteriocins display bactericidal or bacteriolytic properties against closely related species [29,30,31], broader spectrum antimicrobial activity against other species, including multi drug resistant bacteria has been documented [32,33,34]. The best known bacteriocin, nisin A, is produced by the lactic acid bacteria (LAB) *Lactococcus lactis* and is currently approved for use as a food preservative in over 50 countries. Indeed bacteriocins have traditionally been mainly used as food preservatives and probiotics, however, properties such as their potency, low toxicity, the availability of both broad and narrow spectrum peptides and low induction of resistance has led to their increasing recognition as viable alternatives to classical antibiotics [35].

Bacteriocins are divided into two major groups: Class I (modified) and class II (unmodified or cyclic) [36]. In turn, Class I bacteriocins are subdivided into several sub groups comprising a large number of different members including lantibiotics (of which nisin is a member), thiopeptides, sactibiotics, glycocins and modified microcins. The smaller class of unmodified or circular (class II) bacteriocins can be divided into groups that correspond to the four subclasses of unmodified LAB bacteriocins [36].

While they are best characterized in LAB and *Bacillus* species it has been estimated that almost 99% of all bacteria produce at least one bacteriocin, resulting in a vast diversity of compounds with huge potential to be exploited for therapeutic purposes [37]. Recent studies of marine associated bacteria identified bacteriocin-like compounds produced by the genera *Proteus*, *Providencia*, *Klebsiella*, *Alcaligenes*, *Bacillus*, *Enterococcus* and *Cyanobacteria*, all of which displayed anti-microbial activity against both foodborne and animal pathogens [38]. Furthermore, *in silico* analysis of genome and metagenome datasets suggest a widespread distribution of class I bacteriocins including sactibiotics, and lantibiotics [39,40,41].

**Figure 2:**
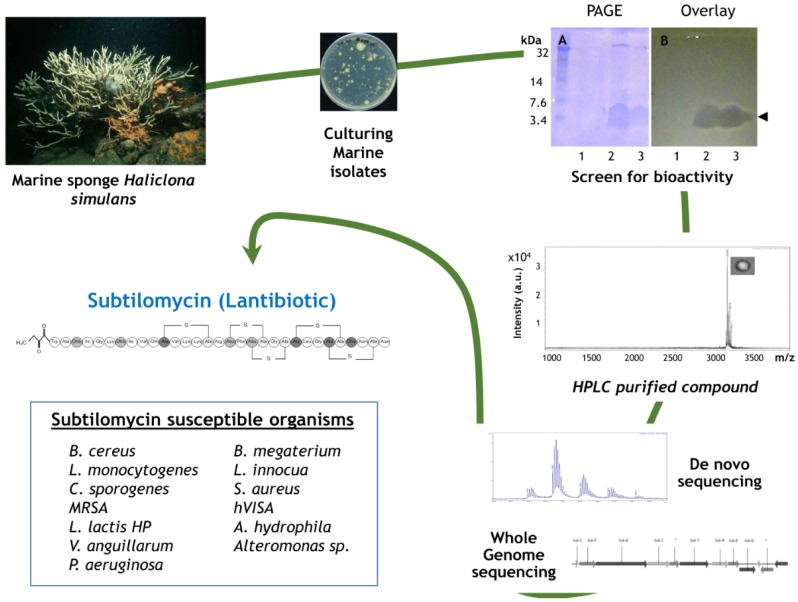
**Marine biodiscovery pipeline for subtilomycin**. The identification and isolation of novel bioactive compounds from the marine ecosystem requires the integration of several technologies. The cross-disciplinary nature of these pipelines merely reflects the complexity of the natural compounds that are produced by marine bacteria and other organisms. Produced by a marine sponge isolate that was found to produce an antimicrobial compound upon initial screening, isolation and characterization of the novel lantibiotic required the combination of chemical and genomic technologies. Subsequently, purified and characterized subtilomycin was found to have activity against a broad spectrum of Gram negative and Gram positive pathogens.

The class I bacteriocins, lantibiotics, are small peptides ranging from 19 to 38 amino acids in length which undergo extensive post translational modification rendering them more stable and resistant to proteolytic degradation, thus offering a significant advantage compared to other standard bacteriocins [36,42,43]. In general, lantibiotics, like nisin, bind to the docking molecule lipid II. Here the peptide induces cell lysis via pore formation, resulting in the decrease of the membrane potential and the efflux of small metabolites from the target cells [43,44]. Recently a novel lantibiotic, subtilomycin, produced by a marine sponge, *Bacillus subtilis*, isolate was identified, which displayed broad spectrum antimicrobial activity against both Gram-positive and Gram-negative bacteria (Figure 2) [45]. Interestingly subtilomycin displayed a lower minimum inhibitory concentration (MIC) towards the Gram-positive pathogen *Listeria monocytogenes*, compared to nisin and possesses several physicochemical properties supporting its potential use in the food or pharmaceutical industry. Interestingly, the subtilomycin biosynthetic cluster is widespread amongst *B. subtilis* strains isolated from different shallow and deep water marine sponges, but it appears to be absent from many *B. subtilis* isolates from other environments. This, allied with its novel structure, suggests that the marine ecosystem could harbor a vast untapped reservoir of these novel activities. More recently, subtilomycin has also been identified in a plant endophytic *B. subtilis* isolate BSn5 where the transmembrane protein ApnI was shown to act as an immunity protein, sequestering the lantibiotic to the cell membrane, in what is a novel model for lantibiotic immunity [46].

### 2.2. Nanoantibiotics

In spite of clear benefits from the use of peptides, currently 95% of peptide properties have limited pharmaceutical applicability, such as short half-life in the circulatory system, toxicity, uncontrolled release, biodistribution, immunogenicity, and costs for therapies [47]. Proteins and peptides are strongly affected by rapid proteolysis in the blood stream and low permeability across biological barriers. Furthermore, physicochemical properties, size, shape, and route of administration, can markedly affect the effectiveness of these potential therapies *in vivo* [48]. Nanobiotechnology has begun to provide solutions to these limitations, leading to the development of what are now called nanoantibiotics [49]. Nanostructures have been considered as efficient carriers since they provide higher levels of biodegradability and biocompatibility [47]. Furthermore, injectable nanoparticles can be driven to different tissues, since they are directly affected by blood vessel endothelium. Clavanins, cationic antimicrobial peptides isolated from the marine tunicate *Styela clava*, have been encapsulated in order to develop nanoantibiotics against bacterial sepsis [47]. The nanostructured clavanin exhibited non-hemolytic partial control of the development of *Staphylococcus aureus*, *Klebsiella pneumoniae*, and *Pseudomonas aeruginosa*. Methacrylate nano-encapsulation of clavanin A provided protection, specificity and controlled release of the peptide. Importantly, the nanoparticles presented morphological characteristics that are viable for injectable drug applications, being non-cytotoxic and of suitably small size, although further experiments are needed to evaluate the use of methacrylate nanoparticles for this purpose. Another study described the design of synthetic analogues of natural antimicrobial peptides. The authors designed a hydroxyapatite/gold/arginine (HAp/Au/arginine) nanocomposite that contains: (a) hydrophobic gold (Au) nanoparticles; (b) positively charged hydrophilic arginine molecules that functionalize the surface of the Au; and (c) hydroxyapatite (HAp) bioactive carrier of the functionalized Au nanoparticles [50]. In comparison to the non-selective HAp/Ag reference, the nanocomposite possessed stronger antibacterial action, was more compatible to human cells and may potentially be a safe and effective replacement for Ag-based antibacterial components in biomaterials.

In addition to providing a structure for modification of existing and new antimicrobials, the marine ecosystem is also a source of nanoparticles themselves. Marine microorganisms such as bacteria, cyanobacteria, yeasts, fungi, and algae have already been reported to synthesize inorganic nanoparticles [51], as have mangroves, salt marshes, sand dunes, and marine animals such as finfish and sponges [51]. However, our understanding of the processes underpinning this phenomenon is currently limited and further research is needed before integrating these nanoparticles into therapeutic development processes.

### 2.3. Peptidomimetics

Protein-protein and protein-ligand interactions form the basis of signaling systems across the kingdoms of life. From simple, on-off sensory systems, to concentration and duration dependent hormonal signaling systems, the nature and mechanism of these interactions is of significant interest. Classically, many of these signaling systems were thought to be insulated through structural specificity and conservation, although more recently we are beginning to understand the inter-relationship and cross-talk that is possible between distinct systems. Notwithstanding this complexity, there are two broad classes of complexes: “domain-domain” in which both components comprise pre-folded structural units, and “domain-peptide” in which one component is a short motif that is unstructured in the absence of its binding partner [52]. Peptidomimetics, compounds which structurally mimic the key binding elements (pharmacophore) of the native peptide or protein and retain the ability to interact with the biological target and produce the same biological effect [53,54], seek to identify and modulate these interactions, and are providing interesting opportunities for therapeutic development [53,55,56,57].

Importantly, peptidomimetics offers a strategy to overcome the issues surrounding the use of peptides in clinical therapeutics. These include rapid degradation by proteases, poor oral availability, difficult transportation through cell membranes, nonselective receptor binding and challenging, multistep preparation [58]. Peptidomimetics have been prepared by cyclisation of linear peptides and/or coupling of stable unnatural amino acids. Amine alkylation, side chain substitution, structural bond extension, cyclisation, and isosteric replacements within the amino acid backbone have all been undertaken to generate unnatural amino acids from their native analogues [58]. Isosteric replacements within a peptide backbone confer diverse electrostatic properties and new secondary conformations on the peptidomimetic chain, often resulting in improved pharmaco-kinetic properties. The diversity of peptidomimetics that are currently available is extensive and includes: α- β- and γ-aminoxypeptides achieved through backbone extension; azadepsipeptides, azatides, azapeptides which are generated through replacement of the α-carbon; as well as thiodepsipeptides and depsipeptides, which are produced via isosteric replacement of the amino functionality.

Peptidomimetic antibiotics based on the antimicrobial peptide protegrin I have shown activity in the nanomolar range against Gram-negative *Pseudomonas* spp., but were largely inactive against other Gram-negative and Gram-positive bacteria [57]. These peptidomimetics also showed potent antimicrobial activity in a mouse septicemia infection model. This approach has also been applied to several members of the conotoxin family, marine toxins produced by the marine snails belonging to the genus *Conus* [53]. A range of peptidomimetic strategies have been employed, including *de novo* design, hybrid design based on anthranilimide and a diphenylmethylpiperazine, truncated analogues rationalized by conformation around the *N*-benzyl moiety, with the ω-conotoxins receiving particular attention. However, to date, the peptidomimetic strategy has proved challenging, with attempts at mimicking the conotoxins with non-peptide molecules generally resulting in significant losses in potency [53]. In contrast, direct modification of the peptide toxins has proven to be more effective, with dicarba and cyclisation approaches resulting in potent and more stable analogues of the native peptides.

In many cases the marine ecosystem provides its own repertoire of natural mimetics. Heparinoids isolated from marine shrimp have presented potent anti-angiogenic properties in both *in vitro* and *in vivo* [59]. Marinopyrrole A (more commonly referred to as maritoclax) was identified from a species of marine-derived Streptomycetes [60]. In addition to exhibiting potent antimicrobial activity against methicillin-resistant *Staphylococcus aureus*, this natural product has also been proposed as a novel class of Mcl-1 inhibitors [60]. However, further evidence for e.g., binding affinities and involvement of the BAX/BAK pathway, are required before maritoclax can be confirmed as a natural BH3 mimetic specific for MCL-1 [61].

### 2.4. Peptide Nucleic Acids

Peptide nucleic acids, first introduced almost a quarter of a century ago, provided a promising platform for the development of new antimicrobial compounds. Based upon the nucleic acid framework, where the sugar-phosphate backbone has been replaced by a synthetic peptide derivative, these molecules have found application in many fields of science from pure chemistry, molecular biology, drug discovery, and (genetic) diagnostics, to nanotechnology and prebiotic chemistry [62]. PNA hybridizes with complementary sequences through complementary base pairing and helix formation. The peptide backbone provides superior hybridization, resistance to enzymatic degradation and access to a variety of chemical modifications. These synthetic constructs have found applications in marine ecology, being particularly effective in monitoring microbial biodiversity and identifying species flux in mixed microbial populations [63,64].

Peptide nucleic acids (PNAs) have also been tested as antimicrobial agents in the past decade in a variety of bacterial species [62,65,66,67,68,69,70,71]. Proven to be very stable in human serum and cellular extracts, as of yet no known nuclease or protease has been shown to be capable of hydrolyzing PNAs [70]. For antisense applications, target bound PNA can cause steric hindrance of DNA and RNA polymerases, reverse transcriptase, telomerase and the ribosome. The mRNA of several essential genes has been targeted by PNA antisense interference to achieve inhibition of bacterial growth and reduced virulence gene expression in a broad range of pathogenic organisms. The antisense peptide-PNAs specifically inhibited expression of DNA gyrase subunit A and OmpA from the respective targeted genes in a dose-dependent manner in *Klebsiella pneumonia* [68]. The stop codon of *S. aureus* has also been targeted effectively, with reduced growth and virulence gene expression observed [69]. PNAs targeted to *Brucella* genes involved in DNA (*polA*, *dnaG*, *gyrA*), RNA (*rpoB*), cell envelope (*asd*), fatty acid (*kdtA*, *acpP*) and protein (*tsf*) synthesis were shown to inhibit the growth of *B*. *suis* in culture and in macrophages after 24 h of treatment [71]. In a different approach, bacterial protein biosynthesis has been inhibited by targeting with PNAs specific for the 16S or the 23S RNA [72].

A general problem with the development of antisense agents is poor cellular uptake. The outer membrane of Gram-negative bacteria can provide impressive resistance against a wide variety of compounds. To overcome this, cell-penetrating peptides (CPPs), which are naturally occurring or synthetic peptides containing positively charged residues that are able to enter eukaryotic cells and bacteria, have been employed [65,66]. Two such CPPs, arginine rich HR9 and IR9, were found to be effective at entering rotifers, small zooplankton that occur in freshwater, brackish and marine environments, thereby having the potential for delivery of exogenous genes, proteins and nanoparticles into these and other eukaryotic systems [73]. Antisense peptide-PNAs (PPNA) have also been shown to be effective against *Escherichia coli*, where delivery was enhanced using attached cationic carrier peptides. The KFFKFFKFFK carrier peptide conjugated to short PNAs (9–12 residues) targeted to the start codon region was found to provide robust antisense effects in this organism [67]. Furthermore, (RXR)4XB- and (KFF)3K-conjugated PNAs were shown to be bactericidal in concentration-dependent and sequence-selective manner, whereas a PPNA with a scrambled base sequence had no effect on growth [65]. Activity was shown against multidrug-resistant *E. coli*, *Salmonella enterica*, *K. pneumoniae*, and *Shigella flexneri in vitro* and *in vivo [65]*. In another study, four antisense PNA oligomers conjugated to the H-(R-Ahx-R)4-Ahx-bala or the H-(R-Ahx)6-bala peptide exhibited complete growth inhibition of *Pseudomonas aeruginosa* strains PA01, PA14, and LESB58 at 1–2 mM concentrations without any indication of bacterial membrane disruption (even at 20 mM), and resulted in specific reduction of the targeted mRNA levels [66]. While growth affecting compounds have obvious potential, antisense-mediated downregulation of virulence-related genes may also provide a therapeutic advantage in that pathogens with attenuated virulence could be targeted by the host immune system leading to a subsequent clearance of infection without the requirement of further antimicrobial treatment. Moreover, in some cases, antimicrobial synergy can be observed upon combined treatment, both dependent and independent of the pathway targeted by the antibiotics [70].

## 3. Marine Peptides and Cell-Cell Communication

### 3.1. Signaling in the Marine Ecosystem

Quorum sensing (QS) is the term used to describe coordinated behavior of bacterial cells through signal production and perception, tightly regulated at the level of a threshold or quorum. Previously thought to be uniquely controlled at the level of cell-density, these systems are now known to be more complex and exquisitely fine-tuned. A diverse range of signaling systems have been described in Gram-negative bacteria, with the broad spectrum acyl homoserine lactones (AHLs) often complemented with species specific systems e.g., the Pseudomonas quinolone signal (PQS) in *P. aeruginosa*. On the other hand, QS in Gram-positive organisms is controlled through the production and perception of auto-inducing peptides (AIPs). Produced as precursor propeptides in the intracellular compartment, these AIPs are further processed by a membrane-bound endopeptidase and secreted to the extracellular environment as mature AIPs. Gram-positive bacterial signaling pathways may be classified into one of four groups with a defining hallmark: Cyclical peptides of the Agr type, peptides that contain Gly-Gly processing motifs, sensory systems of the RNPP family, or the recently characterized Rgg-like regulatory family [74]. However, while QS molecules have been detected in Gram-negative bacteria of marine origin [75,76], QS peptides from Gram-positive organisms of marine origin remain to be discovered.

The diversity of anti-QS molecules produced by Gram-positive marine organisms would suggest that peptide based signaling is present in the marine [10,77,78,79]. Indeed, several characteristics of short peptides make them particularly suited to carry the information between intercellular communication systems. Described previously by Browne and Zimmer-Faust [80] these include: (a) peptides are typically soluble rather than volatile at neutral pH owing to the charged nature of the terminal primary amine and carboxylic acid groups; (b) cells of every order of life are already equipped with the structural units (amino acids), machinery (enzymes), and templates (DNA through messenger RNA) for producing peptides; (c) the diversity afforded to cells in producing short peptides, whereby the degree of uniqueness increases at a rate of 20*n* (where *n* is the polymer chain length); and (d) marine bacteria encode a suite of protease enzymes, both intra- and extracellular, that can rapidly degrade or process peptides, thereby either terminating signal initiation or altering the activity of the signal peptide. This confers an exquisite level of control on cells, providing a mechanism through which to control and influence the growth and behavior of communities in a complex ecological system. However, the isolation and characterization of peptide based QS molecules from the marine remains to be achieved.

The dynamics and networks associated with these QS peptides have been well described in human pathogens such as *S. aureus*. These peptides are diverse in structure and are also amenable to chemical modification, and are therefore excellent platforms for the development of a range of bioactivities. The need for standardization of the spectrum of peptide-associated QS systems in bacteria has led to the development of database systems, e.g., Quorumpeps [64]. This database describes the microbial origin (species), functionality (method, result and receptor), peptide links and chemical characteristics (3D-structure-derived physicochemical properties) of the QS oligopeptides, providing a framework for analysis of marine QS peptides, if and when they are discovered.

### 3.2. Peptides and Microbial Culturability

One of the great microbial anomalies of the last century has been the lack of concordance between the breadth of microbial diversity that exists in the environment and our ability to cultivate and observe this on traditional culture media. Known as the great plate anomaly, anything from 99 to 99.9 percent of microbial diversity has generally been considered to be unculturable, depending on the niche from which it was taken and the degree of investigation and media optimization undertaken for the most dominant species. Genomic technologies have gone a long way towards providing a mechanism by which we can mine these so-called unculturables, with synthetic biology providing a basis for the expression and production of the most interesting bioactivities. However, there remains an unmet need to create the conditions whereby the vast majority of microbial organisms can be cultured.

A range of approaches have been undertaken to achieve this goal, with supplementation proving one of the more successful. Addition of key niche-specific metabolites to the media has led to some advances in the culturability of new organisms, while *in situ* culturing has led to significant discoveries of novel natural products. More recently, the role of signaling molecules and in particular short marine peptides in enhancing the culturability of marine organisms has received attention [17]. This is based on the premise that the signaling networks that are intrinsic to many microbial organisms are simply not present in standard monoculture petri dish based systems. One of the first reports exploring this approach described the impact of cAMP and the homoserine lactone signal molecule on growth of marine organisms [16]. The concept was based on the old premise that the growth of singular organisms within the community depended on the production of molecules or cues by the other members of that consortium. However, rather than providing a nutritional cue or metabolite for growth, these signal molecules are more likely activating conserved signal networks linked to expression of key pathways for growth in receiver organisms.

Previously, a short 8-kDa peptide produced by *Mycobacterium tuberculosis* was shown to resuscitate non-dividing cells [81]. Subsequently, muropeptides produced by the same organism were shown to produce a similar effect [82]. This suggested that marine peptides may elicit an analogous effect in marine communities. Indeed, Nichols and colleagues recently isolated a 24-mer peptide derived from casein and produced by a *Psychrobacter* helper organism, which would appear to facilitate growth of the marine organism through signaling rather than the provision of a nutritional cue [20]. Provision of a synthetic short 5-mer peptide was sufficient to enable growth suggesting that signaling was the basis of the interaction. The growth dynamics of the study involved the process of domestication, whereby growth of the previously uncultivable organism in the presence of a concentration of peptide produced by a helper organism led to the emergence of a small number of cultivable variants in the population, which could then be selected for. This process has emerged from similar studies whereby the environmental isolates are cultured in diffusion chambers under conditions replicating their own environment. After a round of culturing *in situ*, adapted variants can be selected for growth under standard laboratory conditions. This process of domestication is providing access to a wealth of new organisms that can be mined for bioactivities [19].

Another study by Pustam and colleagues revealed how the cationic antimicrobial peptide, protamine, enhanced the growth of *Pseudoaltermonas* sp. in the presence of Mg^2+^ and Ca^2+^ [23]. Cationic antimicrobial peptides are associated with killing of Gram-negative bacteria and enhancement of growth would come as a surprise in this regard. However, the divalent cations would appear to be central to this phenomenon and previous work on microbial pathogens implicated these ions in protection from similar cationic antimicrobial peptides such as colistin. It may be time therefore to consider the physiological role of these peptides in the marine ecosystem, where growth promotion rather than suppression may be a key factor. Interestingly, exposure of *P. aeruginosa* to the cationic antimicrobial peptide colistin led to induction of the central QS system PQS and its biological precursor HHQ [83]. Similar induction in marine organisms in response to peptides may play a significant role in modulating the population dynamics in that ecosystem. Whether or not these interactions are the result of cooperative or competitive interactions remains to be ascertained [18].

## 4. Next Generation Marine Peptides: Anti-Biofilm and Anti-Virulence Bioactivity

Microbial infections remain a major global health issue for medical professionals and public health systems. The rapid spread of antimicrobial resistance across all classes of antibiotics, rendering many conventional antibiotics ineffective, has focused attention on the need for alternative strategies to infection control. During pathogenesis, bacteria employ a vast array of virulence factors to overcome the host defense and establish infection [84]. The process is highly adapted and multifactorial, often requiring the temporal and coordinated expression of genes, either in tandem or in sequence, to successfully colonize the host environment. To add to this complexity, infections are rarely unique, and occur as polymicrobial communities, with interspecies and interkingdom interactions superimposed on the bi-directional signaling between microbe and host [85]. Together, these multiple interactomes constitute what is effectively a chorus of communication, exquisitely coordinated and highly evolved. Understanding how pathogenic bacteria use virulence factors to interact with their hosts and originate the disease is a prerequisite to define new targets for vaccines and drug development.

A key approach that is developing significant potential is the molecular disruption of biofilm formation through the use of peptide or signal mimics. Biofilm formation is a multicellular behavior that is almost universal among microbes, and the distinct stages of biofilm formation are coordinated through signal dependent regulatory systems. This provides researchers with the opportunity to develop smart drugs that intercept the biofilm signal, thus locking the cells into a planktonic, antibiotic-sensitive state. Marine biodiscovery is providing small molecules with the potential to deliver on this next generation approach to the clinical management of infections. Advanced signal dependent screening platforms, linked to molecular biosensors e.g., gene-traps, are underpinning the discovery of new classes of antimicrobial compound. This approach has already led to the identification of a suite of signal-disruptive compounds with biofilm-blocking potential and chemical tractability.

More broadly, targeting QS systems constitutes another novel and emerging pharmacological approach to control bacterial virulence and biofilm formation. As with the anti-biofilm approach, this effectively allows the host defense system to eliminate attenuated bacteria or substantially increase the effect of co-administered antibiotics.

### 4.1. Anti-QS Compounds

The vast repertoire of small molecules encoded in the marine ecosystem has already provided researchers with considerable success in the identification of molecules that interfere with QS in pathogenic organisms [10,77,78,79]. Small aromatics, enzymes, and cyclic peptides have all been isolated and characterized with anti-QS activity, against either Gram-negative or Gram-positive organisms. The targets for QS inhibition are varied among different classes of organism, with peptide inhibitors expected to be most effective against Gram-positive bacteria. The Gram-positive QS system comprises of a two-component membrane-bounded histidine kinase receptor and a responsive regulator. Downstream components of AIP-mediated signaling systems include AIP synthases, efflux AIP transport systems, and transcriptional regulators, all of which are also considered as targets for QS inhibition (Table 2).

Mechanistic information underpinning QS inhibition is limited for many of these studies, although perhaps best characterized is the agr system of *S. aureus*. Several reports have described peptide and non-peptide based compounds that interfere with agr signaling in *Staphylcocci*. For example, Peterson and colleagues reported apolipoprotein B as a sequestrant of AIPs from *S. aureus* preventing the activation of the receptor and therefore the expression of virulence genes [86]. Another study by Otto and colleagues revealed that derivatives of *S. epidermidis* AIPs successfully suppressed virulence linked toxin expression, independent of any growth limitation [87]. Alternatively, studies have targeted virulence systems that interface with the agr system, such as the RNA–III activating peptide (RAP) system in *S. aureus*. This key virulence pathway was targeted by RNA III inhibiting peptide (RIP), a heptapeptide originally isolated from *S. xylosus* [88,89]. Synthetic derivatization of the natural RIP has led to the development of compounds that are capable of reducing *S. aureus* infections such as cellulitis, septic arthritis, keratitis, osteomyelitis and mastitis [89,90]. Furthermore, a non-peptide analogue of RIP, known as 2′,5-di-*O*-galloyl-d-hamamelose (hamamelitannin), a natural product of *Hamamelis virginiana* (witch hazel) has also been shown to be effective in suppressing *S. aureus* and *S. epidermidis* infection [89,91].

Cyclic dipeptides are relatively simple molecules and, thus, have been described to be one of the most common peptide derivatives found in the nature [92]. Diketopiperazines (DKPs) are an interesting family of structurally diverse cyclic dipeptides with promising biological properties. These peptides were isolated from marine sponges more than 30 years ago [93], and later, were also isolated from Gram-negative (*P. aeruginosa*) [94] and Gram-positive (*Micrococcus* sp.) [95] marine sponge-associated bacteria. Subsequently, De Rosa *et al.* (2003) described the production of several DKPs by two bacterial isolates that were identified as strains of the genus *Staphylococcus* and *Bacillus* [96]. These data supported the idea that marine bacteria could be an important source of this class of bioactive compounds. A few years before, Holden *et al.* (1999), identified in the cell free supernatant of *P. aeruginosa* and in other bacteria, two compounds that were able to activate an *E. coli N*-acylhomoserine lactone (AHL) biosensor strain harboring the LuxRI QS system genes cloned in a plasmid [97]. These two compounds were characterized by Mass spectrometry and NMR spectroscopy revealing that they were not AHLs, but they were the DKPs, cyclo d-Ala-l-Val and cyclo l-Pro-l-Tyr. The latter was also produced by the Antarctic sponge-associate *P. aeruginosa* [94] and by Bacillus D28 strain isolated from marine sediment [79]. These two peptides were tested against other QS phenotypes and it was surprisingly observed that the swarming motility of *Serratia liquefaciens* was inhibited and not activated by both. Even more surprising was that even when C4-HSL was added at 150 mM cyclo l-Pro-l-Tyr was still able to inhibit the swarming motility. This cyclic dipeptide isolated from the marine Bacillus D28, also showed antagonistic activity against the QS regulated bioluminescence of *Vibrio harveyi*.

Apart from the cyclic dipeptides, linear dipeptides have also been discovered which showed virulence attenuation of *P. aeruginosa* virulence [98]. In this study, the authors investigated the potential of actimomycetes associated with marine invertebrates as QS inhibitor producers. Of over 72 isolates tested, three *Streptomyces* sp. (NIO 10058, NIO 10068 and NIO 10090) were identified, of which isolate NIO 10068 showed the highest anti-QS activity, inhibiting different virulence factors of *P. aeruginosa*. After ESI-MS analysis of the of NIO 10068 methanol extract, the authors concluded that the isolate produced cinnamic acid and the linear dipeptides Pro-Gly and *N*-amido-α-Pro in the active extract. Independent QS inhibition analysis using the different molecules showed that the QS inhibition activity of the two linear dipeptides was less significant compared with that of cinnamic acid. Furthermore, cinnamic acid could be considered as a peptide mimetic, due to the fact it is the product of the catalysis of L-Phe by phenylalanine ammonia lyase. This was the first study describing anti-QS activity for cinnamic acid isolated from the marine bacterium *Streptomyces* [98].

**Table 2:** Marine microbial-derived peptides against AIP and AHL dependent-QS systems.

Bacterial Species	Isolated From	Peptide Inhibitor	Target QS System and Phenotypes	Source
*P. aeruginosa*	Marine Antarctic sponge	DKP, Cyclic dipeptide: Cyclo l-Pro-l-tyr	Interferes with AHL-QS system	[94]
			Inhibits bioluminescence by *V. harveyi*	
			Inhibits *V. fischeri luxR*	
*Bacillus* sp. D28	Marine sediment	DKP, Cyclic dipeptide: Cyclo l-Pro-l-tyr	Interferes with AHL-QS system	[79]
			Inhibits bioluminescence by *V. harveyi*	
			Inhibits *V. fischeri luxR*	
*Streptomyces* NIO 10068	Marine sponge	Linear dipeptides: Pro-Gly	Interferes with AHL-QS system	[98]
			Inhibition against *P. aeruginosa*: Swarming motility, pyocyanin production, biofilm formation, rhamnolipid production, LasA protease production	
			Inhibits violacein production by *C. violaceum*	
		*N*-amido-α-Pro	Belong to the active fraction, but its effect against QS system was not demonstrated	[98]
*Photobacterium halotolerans*	Mussel surface	Cyclodepsipeptides: Solonamide B	Interferes with the AIP-QS system	[78,99,100]
			Increases *spa* expression	
			Reduces the expression of *hla* and *rnaIII*	
			Interferes with the binding of *S. aureus* AIPs	
		Ngercheumicin F,G,H, and I	Interferes with the AIP-QS system	[77]
			Modulate the expression of QS regulated virulence genes: Increases *spa* expression	
			Reduces the expression of *hla* and *rnaIII*	

More complex peptides, cyclodepsipeptides, have been recently described in a *Photobacterium* strain for their anti-QS activity against *S. aureus* [99,100]. The structures of these two depsipeptides consist of a macrocycle with l-Phe, l-Ala, l-Leu and d-Leu, with a hydroxyoctanoic and hydroxyhexanoic acid in Solonamide B and A, respectively. Although these two structures are similar, just the Solonamide B was described to dramatically reduce the expression of genes involved in the virulence phenotypes controlled by the agr QS system in *S. aureus*, suggesting that the fatty acid chain may play an important role on the anti-QS activity [99,101]. In a later study, Nielsen *et al.* (2014) have demonstrated that Solonamide B also interfered with the binding of auto-inducing peptides (AIPs, QS signal molecules) to the histidine kinase sensor, AgrC, of the agr QS system of *S*. *aureus* [78]. In a separate study, purification of components from the pellet fraction of *P. halotolerans* led to the isolation of ngercheumicin F, G, H, and I [77]. These four new cyclodepsipeptides also interfered with expression of the agr QS virulence regulon of *S. aureus*, although to a lesser extent than the previously described solonamides from the same strain of *Photobacterium* [77]. This suggests that the reservoir of anti-QS peptides may be larger than previously thought in this and other marine organisms.

In addition to peptide based interference with quorum sensing, enzymatic degradation of quorum sensing signals has also received considerable attention. Commonly termed quorum quenching (QQ), these enzymes are grouped in two different classes: (a) those that degrade the signal molecule such as AHL-lactonase and AHL-acylase; or (b) those that target the acyl chain component reducing the carbonyl to hydroxyl without degradation. QQ is likely to be a common activity in marine bacteria because a high abundance of QQ bacteria was found among marine cultivable bacteria and a high frequency of QQ genes was discovered in marine metagenomes. Although reports of AHL-degrading activity of marine bacteria are limited, more than 30 species of QQ bacteria belonging to *Alphaproteobacteria*, *Gammaproteobacteria*, *Actinobacteria*, *Flavobacteriia* and *Firmicutes* have been identified thus far. Furthermore, while targeting QS in pathogenic organisms has proven a successful strategy in controlling virulence behavior, QS-independent suppression of virulence has also received considerable attention [102,103,104]. Bacteria are equipped with diverse repertoires of sensory systems, embedded in the cell membrane as well as cytoplasmic, transducing the external signal to the appropriate adaptive response. In many cases, elicitation of such an environmental or host related response is linked to the production of virulence behavior, secretion of toxins, and general pathogenesis. Although considerable attention has been focused on the activation of these sensory systems, the interaction between signal and receptor is less well defined. Future technology developments will need to focus on delivering systems with which to identify ligands for orphan sensors, thus driving the generation of smart mimics with which to silence virulence and contain bacterial pathogens in the “harmless” state.

### 4.2. Anti-Biofilm Compounds

Despite the fact that in recent years, there has been an increased number of reports describing novel bioactive compounds produced by marine bacteria that target the biofilm formation in bacterial pathogens [91,105,106,107,108], to date, few marine peptides have been explored in depth. The anti-biofilm activity of a small peptide of 14 KDa, isolated from the marine *Bacillus liqueniformis* D1 has been described [105]. BL-DZ1 was shown to exhibit antimicrobial and anti-biofilm activity against the clinical pathogens *Candida albicans* BH and *P. aeruginosa* PAO1, and against the biofouling bacterium *Bacillus pumilus* TiO1 [105]. This small peptide also showed inhibition of preformed biofilms at concentrations equivalent to MIC for planktonic cells, a feature that could play an important and essential role in fighting chronic established infections. In another study, Das *et al.* (2009), described a lipopeptide biosurfactant, produced by the marine derived *Bacillus circulans* D4 strain, with anti-adhesive properties against different opportunistic pathogens [109]. As adhesion (initial attachment) is the first step in biofilm formation, we could hypothesize the anti-biofilm activity of this lipopeptide, although, its effect on preformed biofilms has not been investigated. More recently, the anti-biofilm activity displayed by exoproducts produced by *Pseudoalteromonas* sp. strain 3J6 isolated in the Morbihan Gulf (Brittany, France) [110], has been attributed at least in part to a peptide named alterocin [111].

## 5. Mining in the Informatics Era

### 5.1. Metagenomes and Genomes

**Figure 3:**
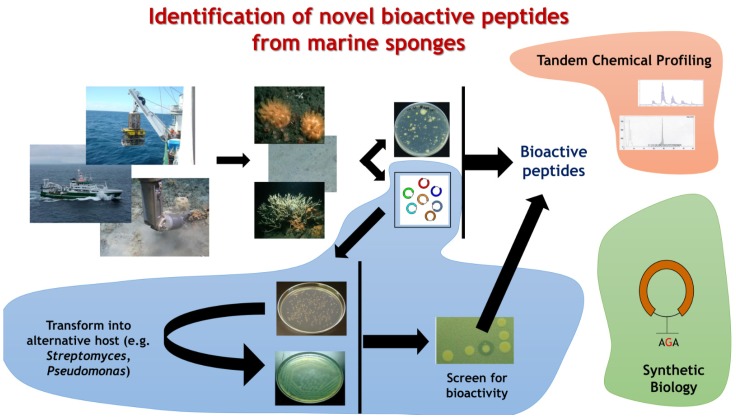
**Marine metagenomics for bioactive peptide discovery**. Overcoming the ‘great plate anomaly’ has proven difficult where marine organisms are concerned and many rare and potentially important bioactivities remain as yet out of reach. However, the advent of metagenomics based technologies has opened up new avenues for exploration and it provides us with a real opportunity to extract new potential from the marine environment. Bottlenecks remain, however, and these will need to be overcome before the full potential of marine biodiscovery can be realized. These include issues surrounding DNA extraction, sequencing depth, heterologous expression, standardization of technologies and metadata, and bioactive detection. Solving these limitations will require the integration of cross-disciplinary expertise backed by powerful data systems and industrial know how.

The explosion in the availability of genomic information has brought with it new insights into the diversity of bioactivities that were previously hidden or out of reach [12,15,112,113,114]. Armed with the genetic blueprint for multiple locations within the marine and indeed other ecosystems, we can begin to infer biological function linked to changes in signature sequences, domains or motifs. Combining genomics, bioinformatics and systems biology, the relatively new discipline of metagenomics has already provided significant advances in our understanding of microbial biodiversity as well as providing access to a rich tapestry of novel bioactivities from bacteria which cannot be cultured using traditional methods [13] (Figure 3). Metagenomics technologies have advanced several research disciplines, including ecology, medicine, and environmental sciences, and the isolation of natural products from previously unattainable sources has proven a major step forward in harnessing the natural potential of the global microbiome.

From a functional perspective, metagenomic libraries allied with innovative chemistry and state of the art robotics, provide an efficient screening mechanism to identify and isolate rich bioactives from the marine and other environments. Access to the culturable and non-culturable resources has significantly enhanced our capacity to mine the oceans for novel and effective antimicrobials, anti-cancer compounds, enzymes and biocatalysts. In addition, the study of metagenomes has greatly advanced our understanding of marine ecology, and particularly the dynamics that underpin community structure in this rich environment. Beginning with the discovery of the Sargasso Sea’s unprecedented diversity of bacteria and functional genes, the abundance of metagenomes currently available has since led to the identification of habitat specific fingerprints. Comparative analysis of these datasets has uncovered correlations between functional metagenome diversity and specialized conditions of environmental niches, a feature that is beginning to inform the functional screens that seek to isolate bioactivities from these ecosystems. However, the continued growth in the number and diversity of metagenomic studies, allied with the significant advances in sequencing technologies have brought with them a global appreciation of the challenges that now bottleneck future developments in this research area [11,13,112,115,116,117,118,119]. Some of these challenges are outlined in Table 3.

**Table 3:** Key considerations for the application of metagenomics technologies.

Metagenomic Challenges	
**When and where to sample**	With the dynamic nature of population flux already reported, where the isolation of novel bioactive natural products is the ultimate goal, the source of the metagenomic DNA is a central consideration.
**Ability to isolate DNA from these samples**	Something that has proven a major bottleneck to complete coverage of library construction and associated screens. The diversity of organisms present, the extent to which they will yield their DNA using conventional or adapted isolation protocols, the differences in abundance between the dominant potentially uninteresting species and the rare potentially lucrative organisms, all present a major headache that needs to be overcome if we are to maximally exploit this technology.
**Size and complexity of current metagenomic datasets**	This presents an additional challenge to researchers with computational advances now urgently required to meet the explosion in available data. Automated genome mining tools and eventually pattern recognition based algorithms are required to deal with the large datasets emerging from these studies. This is crucial in overcoming the oversampling of abundant organisms with loss of information from the lower abundant species.
**Inability to taxonomically link bioactivities to producing organisms**	Perhaps an obvious limitation arising from the heterogeneous nature of microbial communities and the fragment sizes that classically populate metagenomic libraries.
**Expression of eDNA in heterologous hosts**	Even if bottlenecks in sequencing and bioinformatics are overcome, heterologous expression, although possible, is fraught with limitations, including codon usage, rare tRNAs, promoter recognition, toxicity, yield and stability.
**Cryptic or silent bioactive gene clusters**	Activating silent or inactive biosynthetic clusters remains a major challenge, with significant bioactive potential ‘locked in’ within the marine microbial community.
**Standardization of cluster metadata**	The exponential increase in sequence data requires the urgent development of unified standards for the cross-community annotation and description of biosynthetic gene clusters.

There is also an urgent need for sequencing platforms that provide greater depth, with parallel developments in technologies that remove redundant DNA. In spite of the advances arising from next generation sequencing platforms, the short nature of the reads presents its own difficulties, particularly when we consider the large size of many biosynthetic clusters. There is also an urgent need for the development of heterologous expression systems that can elicit activity from silent or cryptic gene clusters. Some success has already been achieved in this regard, but further advances are needed [120,121,122,123,124]. Furthermore, standardization of bioactive gene cluster isolation methods and classification are underway, with the development of bioinformatics tools such as AntiSMASH, and community driven platforms such as MIxS and MIBiG providing excellent frameworks [125,126]. This will significantly enhance the harmonization of metagenomics exploration, providing structure and improved annotation to existing and future genomic, metagenomic, and bioactive gene cluster information.

Metagenomics has also provided a platform for detailed biologically driven analysis of niche-specific signatures. The comprehensive datasets allow us to better understand the distribution of peptide motifs or domain structures within proteins across diverse ecosystems, particularly where conservation of motifs underpins biological activity and biotechnological potential. An example of this is found in both the secretion systems that deliver bioactive molecules out of the cell, and to the secreted compounds themselves, whether peptide or small molecule. Although the number and class of secretion systems are not universal among bacteria, new paradigms for understanding their role in microbial dynamics are emerging. Recent evidence of niche specialization and peptide motif conservation among Type Six lipase effectors in metagenome sequences underpins the need for future structural and bioactivity studies that focus on peptide domains [127]. The differing distribution patterns of the Tle superfamily in various niches are an interesting observation that offers insights into bacterial activity within that niche. Moreover, the differential abundance of Tle families within certain niches as well as the presence of different Tle families within the same genomes suggests that there is specialization of the various Tle families [127]. It is feasible to suggest that this motif specialization will extend to other systems in the marine ecosystem, and can be expected to include the bioactive gene clusters that are described in this review. Motif specialization would be expected to influence the spectrum and activity of these bioactives and uncovering the extent of such specialization will inform future marine mining programs.

### 5.2. Single Cell Genomics

A related technological advance has seen the introduction of single cell genomics to the biodiscovery toolkit, reviewed recently [128,129]. Offering an insight into the heterogeneity that is known to exist in populations, sequencing either of DNA or RNA at the single cell level presents us with the opportunity to dissect the exquisite individualities that exist at the cellular level, while also providing a platform with which to understand the bigger picture at the species or community level. Cell to cell variability is known to exist at the DNA level with acquisitions and loss of foreign DNA accompanied by mutations and deletions made possible with each round of cell-division, albeit with relatively low frequency. Heterogeneity is even more evident at the transcriptional level with growing evidence for cell-to-cell variations, even where the population is phenotypically “homogeneous” [130]. This emerging technology has already found applications in marine diversification studies [131], investigations into organismal interactions [132], and studies on eukaryotic microbial biodiversity [133]. Access to sequencing information at the cellular level will open new avenues for exploration of microbial diversity, and may also identify previously hidden bioactives produced at low abundance within populations, bioactives that would previously have been inaccessible through conventional sequencing approaches [11,134]. Already, we have seen success in this sphere whereby coupling of single cell genomics with metagenomics screening led to the identification of the *apr* gene cluster encoding the apratoxin A anti-tumor compound [135]. Future advances promise combined technologies for the parallel analysis of epi(genomes) and transcriptomes [128]. Furthermore, integration of sequencing and informatics solutions with single cell genomics and transcriptomics technologies will elevate our capacity to decipher the functional capacity of cells, particularly from within mixed microbial populations.

### 5.3. Chemi-Informatics

The use of information based systems to both identify and design the next generation of antimicrobial bioactive peptides has gained significant momentum in recent years, buoyed by the exponential increase in available data. Integrated with functional assessment of activity, quantitative structure-activity relationships (QSAR) are increasingly being used to relate quantitative properties of a compound (known as descriptors) with other properties, such as drug-like activity or toxicity [136,137]. QSAR has become an integral part of pharmaceutical drug discovery pipelines and has more recently been applied to toxicological studies. QSAR analysis has two primary aspects: (a) the choice of the set of descriptors and (b) the choice of statistical learning techniques. QSAR analysis of antimicrobial peptides has previously been limited to comparisons between highly similar peptides, for example, derivatives of lactoferricin and protegrin and similar *de novo* peptides [136,138,139,140]. However, more recently, combinations of QSAR and machine learning techniques have led to the discovery of peptides with enhanced activities relative to the parent molecule. Fjell and colleagues built artificial neural network models using QSAR descriptors on the basis of initial high-throughput measurements of activity of over 1400 random peptides [136]. Subsequent screening of an *in silico* library of approximately 100,000 peptides showed 94% accuracy in identifying highly active peptides. Another approach is the use of so-called matched molecular pairs (MMPs). Such an approach allows identification of molecular transformations that affect particular activities (e.g., toxicity). In contrast to QSAR, chemical interpretation of these transformations is straightforward. Sushko and colleagues have proposed combining the QSAR and MMP approaches by finding MMP transformations based on QSAR predictions for large chemical datasets [141]. The optimization of these systems is ongoing and will interface with and benefit tandem developments in the isolation of bioactive peptides.

### 5.4. Tandem Chemical Profiling

Marine biodiscovery faces several challenges where the characterization of novel compound structures using a chemical approach remains a major bottleneck, limiting our capacity to uncover the next generation of bioactive molecules. The extreme conditions found in marine environments promote microorganisms to produce a diverse array of secondary metabolites, including marine peptides, with a high potential for the pharmaceutical sector [142]. The chemical nature of these bioactive compounds can be extremely complex, making it difficult to follow specific extraction protocols in order to obtain biologically active and pure compounds. In addition, many rare bioactive producing organisms are of significant interest, yet our inability to culture them effectively is an issue. In order to overcome these issues, new and effective methods for peptide extraction are required.

Organic extractions from marine bacteria typically result in complex mixtures of bioactive compounds, requiring downstream separation to isolate the active fraction. A broad separation of the extracts can be resolved by successive extractions using different organic solvents (ethanol, methanol, hexane, *etc*.). The different fractions obtained from different solvents can then be tested using appropriate biological assays (e.g., testing the inhibition of pigment production in the QQ biosensor reporter strains). At this stage, further purification can be achieved using additional chemical separation techniques. In this sense, the chromatographic techniques that are a physical separation method for the characterization of complex mixtures play an essential role in order to eliminate those compounds with no interesting activity. Of these, Thin Layer Chromatography (TLC) and High Performance Liquid Chromatography (HPLC) are the most widely used techniques. Different fractions from the HPLC can be analyzed to identify the biologically active fraction, removing more background compounds. The Preparative Thin Layer Chromatography (pTLC) can be used for the same purpose.

Once the original complex sample has been fractioned and cleaned, more powerful analytical chemistry techniques have to be performed in order to elucidate the chemical structure of the novel bioactives. In this sense, the Mass Spectrometry (MS) and Nuclear Magnetic Resonance (NMR) techniques offer a wide range of possibilities. Using the data of the m/z from the MS, and together with the use of the different NMR techniques (H-NMR, C-NMR and more recently High-NMR), these technologies can offer high resolution in predicting the structural composition of the novel molecules. In addition, the combined use of these techniques with HLPC in tandem or in triplet (HPLC-MS, HPLC-MS/MS or more recently HPLC-MS-NMR) has been demonstrated to be highly effective in establishing the structure of new bioactives from complex samples. Parallel developments in bioinformatics have resulted in a new discipline of natural product peptidogenomics (NPP) [143,144]. This new MS-guided genome-mining methodology iteratively matches *de novo* tandem MS (MS(n)) structures to genomics-based structures following biosynthetic logic [143]. From this, researchers can connect the chemotypes of peptide natural products to their biosynthetic gene clusters. Indeed, the association between tandem mass spectra of natural products and the gene clusters responsible for the biosynthesis of the corresponding compounds has so far proven difficult to establish. Medema and colleagues have introduced a new software package based on Bayesian probabilistic matching to overcome this bottleneck [144]. Along with other developments in the field, Pep2path is designed to optimize the pathway towards high-throughput discovery of novel peptidic natural products.

Therefore, an increased focus on the development of new technologies regarding isolation and separation methods, and more importantly analytical chemistry techniques, is necessary to maximize our capacity to mine the predicted large number of novel marine bacteria with promising activities that have not been identified to date. Together with the extensive amount of genetic information already available in a number of databases, this will significantly enhance the characterization and identification of new natural peptides and the gene clusters involved in their production.

### 5.5. The Advent of Synthetic Biology

Synthetic biology has opened new horizons for the development of improved peptide based molecules. Synthetic molecules that mimic marine products, engineered hosts for natural product expression, and modified antimicrobial peptides are some of the pioneering advances in this exciting new era of biodiscovery. Typically involving the engineering and manipulation of existing products using genetic or chemical approaches, synthetic biology has already led to several successes [11,145,146,147]. Some of the most widely cited are the development of artemisinin, a botanical anti-malarial compounds produced by the wormwood *Artemesia annua*, and the production of taxol, both of which are achieved by the re-constitution of biosynthetic pathways in engineered hosts [146,147]. More recently, there has been a strong emphasis in the integration of synthetic biology with marine biodiscovery, and particularly in the enhancement of the products of bioactive gene clusters. In many cases, new natural products are effectively lead structures, which subsequently undergo synthetic development to improve activity, pharmacodynamics and pharmacokinetics, turning natural products into clinically effective drugs. In addition to the modification of existing natural products, and the engineering of heterologous expression hosts, more recently the advent of “synthetic metagenomics” has received considerable interest [12]. This involves codon optimization of genes of interest followed by chemical synthesis, cloning and expression in an optimized heterologous host. Although an important addition to the biodiscovery toolkit, synthetic biology brings its own challenges and many ethical issues remain to be resolved [148].

## 6. Conclusions

We are in an exciting period for natural product discovery and the potential societal impact of enhanced discovery of novel peptides from the marine ecosystem is only beginning to be understood. Technology developments in this and other fields are converging towards the provision of pipelines for peptide discovery, harnessing expertise in functional genomics, cell-cell signaling, nucleic acids extraction, heterologous expression, bioinformatics, taxonomy, chemical profiling and many others. Keeping pace with these developments are the new applications for marine peptides and their usefulness in the disciplines of peptidomimetics, nanoantibiotics, and as next generation antimicrobials, silencing the virulence and pathogenesis of harmful bacteria. Harmonization of biological, chemical and informatics technologies is key to the success of marine peptide biodiscovery, and will underpin our ability to reach the rare and currently unattainable from the oceans vast reservoir.
